# Effect of Cold Plasma on the Penetration Depth of AH26 and Beta Resin Sealers in Dentinal Tubules

**DOI:** 10.30476/dentjods.2023.98146.2059

**Published:** 2024-12-01

**Authors:** Bahareh Johari, Solmaz Araghi, Mohammad Vahedi, Seyedeh Saba Sharifzadeh, Arash Azizi

**Affiliations:** 1 Private Dentist, Tehran, Iran; 2 Dept. of Endodontics, Faculty of Dentistry, Tehran Medical Sciences, Islamic Azad University, Tehran, Iran; 3 Dept. of Oral and Maxillofacial Medicine, Faculty of Dentistry, Tehran Medical Sciences, Islamic Azad University, Tehran, Iran

**Keywords:** Cold plasma, Dentin permeability, Epoxy resin, Sealer

## Abstract

**Statement of the Problem:**

The creation of a proper seal of the root canal with canal-filling materials, such as gutta-percha and sealer, is one of the essential factors in the success of root canal treatment. In addition, the penetration depth of the sealer is one of the influential factors in creating a proper seal, which improves the sealing ability of the canal and the burial of microorganisms.

**Purpose:**

This study aimed to investigate the effect of cold atmospheric plasma on the depth of tubular penetration of two types of resin sealer: AH26 and Beta RCS sealers.

**Materials and Method:**

In this experimental study, thirty-two premolar teeth with single root and single canal were selected as samples and after cutting their crowns from the apex distance of 15mm, the canals were prepared with rotary files. Samples were divided into four groups of eight, according to the type of sealer and plasma application: AH26 sealer (AH), plasma+AH26 sealer (PAH), Beta RCS sealer (Beta), and plasma+Beta RCS sealer (PBeta). The cold lateral condensation technique was used for the obturation of canals. The maximum penetration depth and the percentage of sealer penetration were obtained from microscopic images at three coronal, middle, and apical sections. Due to the non-normal distribution of data, the Mann-Whitney U test was used for statistical analysis (*p*< 0.05).

**Results:**

No significant difference was observed between the study groups in the penetration percentage and maximum penetration depth of AH and Beta in the presence and absence of plasma. However, in the coronal section, the depth of maximum sealer penetration was significantly higher in the AH group than in the Beta group (*p*< 0.05).

**Conclusion:**

The use of plasma did not affect the maximum penetration depth and penetration percentage of AH26 sealer and Beta RCS sealer.

## Introduction

One of the central factors in the success of root canal treatment is creating a proper seal of the root canal with canal-filling materials, such as gutta-percha and sealer [ 1 ]. Most root canal treatment failures are caused by the persistence of microorganisms in the apical part of the root canals of obturated teeth [ 2 ]. A suitable seal entombs the remaining bacteria in the dentinal tubules and acts as a barrier against the entry of microorganisms through microleakage [ 3 ]. In the absence of a proper seal, some bacteria, such as *Enterococcus faecalis*, can form a colony in the dentinal tubules and lead to the return of the dental infection [ 4 ]. One of the significant factors in the ability to create a proper seal in the root canal is the penetration depth of the sealer, since increasing the contact surface between the sealer and the dentin tissue improves the sealing ability of the canal [ 5 ]. The penetration of the sealer improves the filling of the canal, creates a physical barrier, and causes microorganisms to be buried [ 6 ]. As a result, solutions that cause a greater depth of sealer penetration in the dentinal tubules lead to better canal flooding and allow root canal treatment with a better prognosis. A previous study [ 5 ] investigated the effect of plasma on the depth of penetration of two types of resin and bio ceramic sealer in dentin tubules. The findings revealed that plasma is not effective in the depth of penetration of resin and bio ceramic sealers, although, in comparison, the depth of penetration of bio ceramic sealer was significantly higher than that of resin sealer after plasma application [ 5 ]. Another study examined the effect of plasma on the depth of penetration of two types of resin sealer and bio ceramic, revealing that the effect of plasma on the depth of penetration was negative on the resin sealer and positive on the bio ceramic one [ 7 ]. The present study aimed to investigate the effect of non-thermal atmospheric plasma on the depth of tubular penetration of two types of resin sealer: AH26 and Beta RCS sealer (made in Iran and recently entered the market).

## Materials and Method

**Figure 1:**
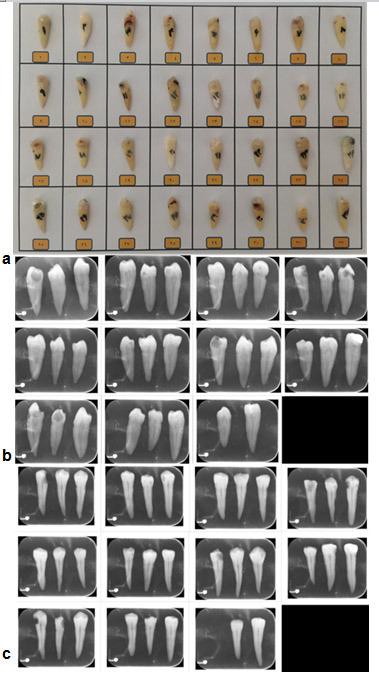
**a:** Samples, **b** and **c:** Bucco-lingual and Mesio-distal radiographs of the specimens, respectively

In this experimental study, the implementation and approval were obtained from the Ethics Committee (IR.IAU.PS.REC.1400.228 ID). Purposive sampling was used to choose the samples based on the following inclusion criteria: single root, single canal, the straightness of the root, and the closure of the root apex [ 5 , 7 ]. On the other hand, samples with root fractures, root resorption, any calcification, and open apex were excluded from the study [ 7 ]. Digital periapical radiographs of the mesio-distal and bucco-lingual views of the samples confirmed that the samples were single canal and that there was no pathological analysis [ 8 ] (Figure 1).

According to the previous study, statistical analysis and using advanced repeated measure ANOVA option of PASS II software, considering α= 0.05, β= 0.2 and effect size= 1.2 for the repeated variable and 0.55 and 0.61 for the two between subject variables, the minimum sample size of 8 were considered as each subgroup of the study and the total number of 32 single-rooted premolar teeth that were recently extracted were selected as samples [ 5 ]. They were kept in 10% ethyl alcohol solution until the samples were completed, and before starting the experiment, they were immersed in 1% hypochlorite solution for four days to remove the debris from the outer surface of the teeth [ 9 ]. The crowns of the teeth were cut so that the length of all roots was 15mm [ 5 ]. The patency of each canal was checked with file number 10 (Mani, Tochigi, Japan), and the working length was considered 1mm shorter than the apex. The initial file (IF) of all teeth was file No. 15 and canals were prepared with rotary files by the Super Files III (Denco, Shenzhen, China) with the single technique length from number S1 to F3[ 7 ]. Between changing each file, 1ml of sodium hypochlorite 6% ADS (Avant Dental Supply Co., Florida, USA) was used. In the final irrigation, to remove the mineral and organic parts of the smear layer, 3ml of 17% EDTA (Asia Chemi Teb Co., Tehran, Iran), 3ml of 6% hypochlorite for, and finally, 5ml of saline were used to irrigate, each for 1min.

**Figure 2:**
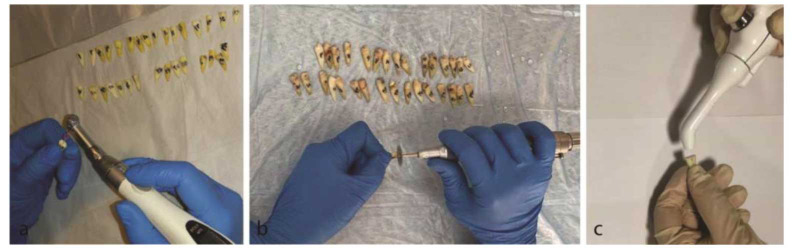
**a:** Cutting the crown of samples from 15 mm to Apex, **b:** Preparation of samples with rotary, **c:** Use of plasma device

Tribest (Tribest Dental Products Co., Jiangsu, China) double-sided cleaning needle was used in all cleaning steps. The canals were dried with a paper point [ 10 ]. The duration of plasma application was considered 30 sec, and the samples were divided into four groups of eight using a simple random method with the Rand option of Microsoft Excel software [ 5 ] (Figure 2). 

In AH26 group (AH), the canals were filled using meta gutta-percha (Metabiomed co., Chungcheong Bukdo, Korea, gutta-percha 2%), master apical cone #35 (MAC 35) and lateral cone #15, #20, AH26 sealer (Dentsply, Konstanz, Germany) and spreader B (Mani, Japan). In Plasma+AH26 sealer group (PAH), the canals were filled with gutta-percha and AH26 sealer after using plasma for 30 sec. In Beta RCS sealer group (Beta), the canals were filled with gutta-percha and Beta RCS sealer (Beta Dandan Maku Co., Maku, West Azerbaijan, Iran). In Plasma+Beta RCS sealer group (PBeta), the canals were filled with gutta-percha and Beta sealer after using plasma for 30sec. 

In PAH and PBeta groups, plasma was applied using the Plasm Art device (Nariatech, Tehran, Iran) in which gas flow is converted into cold plasma by ionization in a dielectric chamber and helium is used as a consumable gas with an input pressure of 4/5 bar. The output pressure of the handpiece is atmospheric. In this study, the distance between the device head and the sample was 5mm, and the gas flow rate was set to 1.85Cm3/s. The power of the device was 8 watts, and the frequency of the cold handpiece was 100 kHz. Plasma flame was applied to each sample for 30 sec with a length of 20 mm and a diameter of 2.5mm. The sealers were mixed according to the manufacturer’s instructions, and a 0.1% solution of rhodamine biofluorescent dye (Merck, Germany) was added to the sealer for confocal microscope analysis. It was then taken into the canal by Lentuo, and canals were filled with gutta-percha, as well as the sealer and cold lateral condensation technique. The canal opening was filled with Cavisol (Golchai co., Alborz, Iran) temporary restorative material [ 5 ]. 

**Figure 3:**
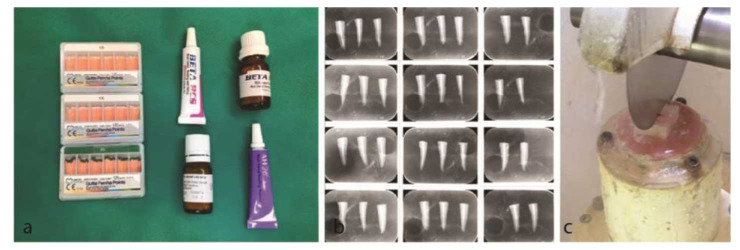
**a:** Obturation materials, **b:** Radiography of obturated samples, **c:** Cutting the specimens mounted in acrylic resin

To complete the set of sealers, the samples were kept for seven days at 37°C in the IM55 incubator (Modern Teb co., Tehran, Iran) [ 11 ]. After seven days, they were vertically embedded in self-curing acrylic resin. Cuts were made perpendicular to the longitudinal axis of the tooth at intervals of 2, 5, and 8 mm from the apex, using a slow-speed water cooled diamond saw (Buehler Isomet 2000, Lake Bluff, IL, USA) equipped with cooling, to obtain pieces with a thickness of 1±0.1mm [ 5 ]. The obtained pieces were representative of the coronal, middle, and apical parts of the root, respectively (Figure 3).

**Figure 4:**
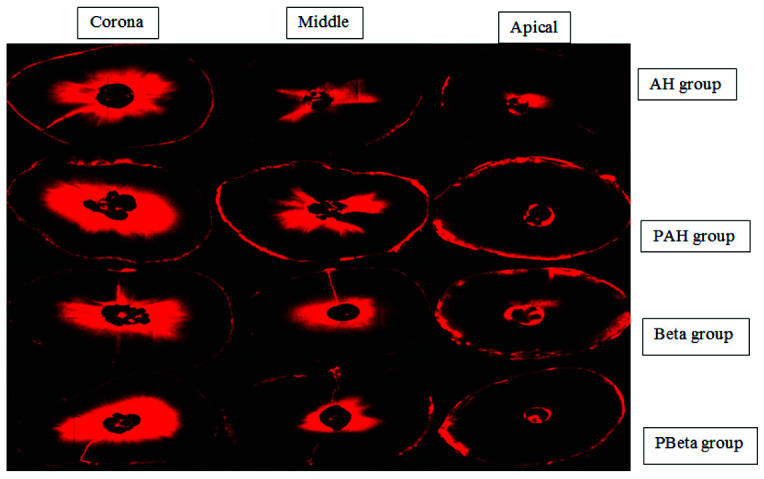
Confocal microscope images of coronal, middle, and apical sections of the studied groups

The coronal surface of the samples was manually polished with 1200, 2400, and 4000 wet silicon carbide abrasive papers, each for 1 min [ 7 ]. Samples were observed with 10 times magnification using Leica TCS SPE confocal microscope (Leica, Mannheim, Germany). Maximum penetration depth and penetration percentage were quantitatively measured [ 5 ] (Figure 4).

The mean and standard deviation of the data were checked using the SPSS software 18 (Version 18.0. Chicago: SPSS Inc), with a significance level of 5% and a confidence interval of 95%, and the normality of data distribution was checked by the Kolmogorov-Smirnov test. Due to the non-normal distribution of the data, the statistical analysis of the data was performed by the Mann-Whitney U test, considering two different types of sealer and using plasma as an intermediary factor with a significance level of 5% [ 5 ].

## Results

**Figure 5:**
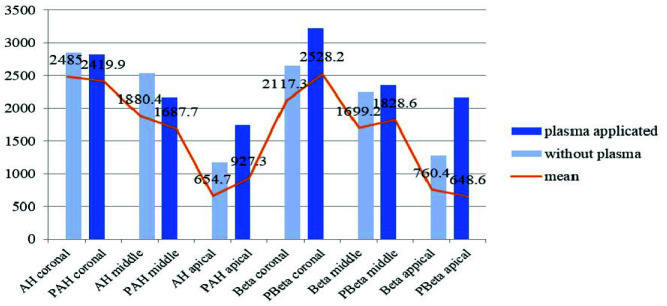
Maximum penetration depth and the average maximum penetration depth in the studied groups in coronal, middle and apical sections

The maximum penetration depth in the coronal section of the PBeta group was higher than that in all the other groups. Moreover, the average maximum penetration depth in this group was higher than that in the other groups, and the lowest average maximum penetration depth among the study groups belonged to the PBeta apical group. The comparison of the two groups with the same sealer (one group under plasma irradiation and another group without irradiation) showed the biggest difference in the maximum depth of penetration was between the Beta apical and PBeta apical groups (Figure 5).

**Figure 6:**
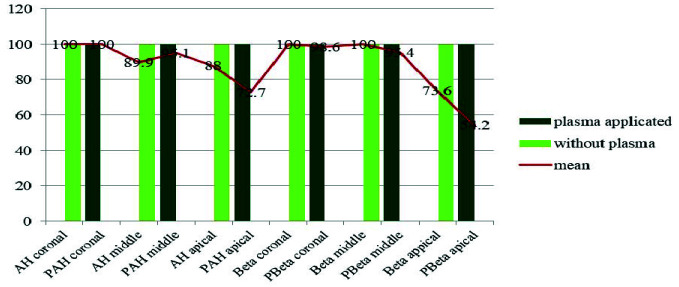
Maximum penetration percentage and average penetration percentage in the studied groups in coronal, middle and apical sections

As can be seen in Diagram 2, the average penetration percentage is 100% only in the AH coronal, PAH coronal, and Beta coronal and Beta middle groups. In addition, the lowest average penetration percentage belongs to the PBeta apical group (Figure 6).

In the comparison of the maximum penetration depth and sealer penetration percentage between different groups of the study in three coronal, middle, and apical sections, the difference was significant in the penetration depth (*p*= 0.038) between two groups of AH and Beta only in the coronal section, and no significant difference was observed in the penetration percentage (*p*> 0.05).

## Discussion

In recent years, the depth of the sealer penetration in dentin tubules has been investigated. Deep penetration of the sealer is necessary because it increases the contact surface between root-filling materials and dentin and improves the quality of sealing [ 12 ]. This arrangement prevents the penetration of bacteria into the dentin tubules. Bacteria that penetrate deeply into dentinal tubules are usually facultative anaerobes. Sealer can trap and inactivate these bacteria and thus plays an important role in healing periapical lesions [ 12 ]. On the other hand, the penetration of the sealer into the dentin tubules can improve the connection between the sealer and the dentin [ 7 ]. Therefore, the deep penetration of the sealer into the dentinal tubules and the amount of tubular penetration of the canal are important factors in the success of root canal treatment. The present study attempted to investigate the effect of a new technology of plasma on the depth of the sealer penetration in dentinal tubules. Several different factors are effective in the penetration depth of the sealer in the dentinal tubules, such as the removal of the smear layer, the diameter of the dentin tubules (which determines the permeability of the dentin), root canal dimension, and the fluidity of the sealer, which depends on the consistency of the ingredients, the size of the particles, and film thickness [ 12 ]. In the present study, Beta sealer, a newly introduced resin sealer, was compared to AH sealer which showed favorable results in different studies [ 13 ]. In this study, a confocal microscope was used to investigate the sealer penetration depth in the samples, which has some advantages over the scanning electron microscope. Some of the disadvantages of scanning electron microscope include the need for gold coating the samples, the harder detection of the sealer in dentinal tubules, the higher magnification of the microscope, and the difficulty to obtain an image of the entire surface. On the other hand, the advantages of confocal microscope include preparing images from different depths of the samples and combining them to create the final image, no need to dry the samples, as well as a higher image resolution and contrast [ 14 ]. Rhodamine B dye with a concentration of 0.1%, which was mixed with the sealer due to its fluorescence properties, did not change the physical properties of the sealer [ 15 ]. Cold plasma is a mixture of ionized gas at room temperature and atoms, as well as molecules, that are free radicals and highly reactive. The energy of these particles is absorbed by solid surfaces that plasma collides with. Moreover, these particles can react with the environment they hit and cause the production of chemical groups on the surface, which helps to increase the adhesion strength of adhesive materials [ 5 ]. Studies have reported the efficacy of a plasma jet against biofilms inside the root canal, which suggest that this technology can be helpful as an adjuvant in conventional disinfection therapies. One of the advantages of plasma is its capacity to reach microorganisms lodged in narrow niches. Although plasma has exhibited favorable antimicrobial properties due to the production of reactive oxygen species, there is little information about its effect on the depth of sealer penetration [ 7 ]. The findings of a study by Sahyon *et al*. [ 16 ] revealed that plasma can improve the surface properties of dentin, wet ability, surface energy, and hydrophilicity. It was also found that regardless of the gas composition, the time of application, and the distance from the sample, plasma does not change the surface hardness, and chemical composition of dentin. Plasma increases the hydrophilic property and surface energy due to the removal of hydrocarbons from the surface and the creation of hydroxyl groups [ 17 ]. Plasma also increases the carbonyl group and decreases the contact angle on the surface, both of which increase the hydrophilicity of the surface [ 5 ]. Another study by Koban *et al*. [ 18 ] showed that the surface molecules are broken through the bombardment of energetic ions, ozone, UV rays, and radical reactions between molecules. The combination of these molecules with atoms or molecules in the air (such as oxygen) increases the surface energy [ 18 ]. The use of plasma causes the drying of the dentinal surface and overlapping of the collagen network [ 19 ]. Only two studies have investigated the effect of plasma on the penetration depth and percentage of sealer penetration. In the first study, Menezes *et al*. [ 7 ] investigated the effect of plasma and photodynamic therapy on the penetration depth and adhesion of MTA-Fillapex ceramic sealer and AHPlus resin. The samples were divided into control, photodynamic, and plasma groups. In the AHPlus resin sealer group, the maximum depth of penetration was higher in the control group than other groups, and the percentage of penetration was similar in all groups. In MTA-Fillapex ceramic sealer group, the penetration percentage and the depth of the sealer had better results in the plasma group than in other groups. However, there was no significant difference in the apical section of both types of sealer. It seemed that the better penetration of MTA-Fillapex, compared to AHPlus, was due to the lower viscosity, higher fluidity, and the ingredients of the MTA-Fillapex sealer [ 7 ]. In the present study, the use of plasma did not significantly affect the percentage of sealer penetration in any of the coronal, middle, and apical sections of the studied groups, as opposed to the above-mentioned study, in which it had a negative effect on the penetration depth of resin sealer. Perhaps the different result can be attributed to the longer use of plasma (one min) in the above study and the drying and overlapping of more collagens as a result of the negative effects of plasma [ 19 ]. In the second study, Gunes *et al*. [ 5 ] investigated the effect of cold plasma on the penetration depth and penetration percentage of AH-plus resin and Endo Sequence BC bio ceramic sealers. There was no significant difference in the percentage of sealer penetration of the studied groups when plasma was employed. Moreover, the use of plasma did not have a significant effect on the penetration depth of resin and bio ceramic sealer, although the maximum depth of sealer penetration in the Endo Sequence BC+plasma group (BCP) was statistically higher than that in the AH+plasma group (AHP). It seems that the AHPlus resin sealer penetrates the micro-irregularities of the canal due to its creep characteristics and high polymerization time and forms covalent bonds with the opened rings of the epoxide sealer wherever collagen amino groups are available. Plasma causes the collagen network to collapse, which may explain the lower maximum penetration depth of the AHPlus resin sealer (numerically but not significantly). On the other hand, plasma generally decreases the contact angle, and argon plasma increases the hydrophilic property of dentin by increasing the carbonyl group. These changes may have increased the maximum penetration depth of the Endo Sequence BC bio ceramic sealer, which is a hydrophilic sealer [ 5 ]. According to the findings of Strazzi-Sahyon *et al*. [ 16 ], plasma does not affect the morphology of dentin tubules. However, other studies showed that it causes the hydrophilic property of dentin [ 17 ] and causes the collagen network to overlap [ 19 ]. It may be concluded that the combination of positive and negative effects of plasma on the penetration of resin sealers means that plasma does not have a significant effect on the depth of penetration of resin sealers in the dentinal tubules. There is a need to conduct more studies on the effects of plasma on the structure of dentin and the penetration depth of resin sealers. In the present study, the depth of maximum sealer penetration was significantly higher in AH groups than in Beta groups in the coronal sections, and there was no significant difference in the depth of the sealer penetration in the coronal sections between PAH and PBeta groups. It seemed that AH sealer is superior to the Beta sealer due to its physical and chemical properties (including fluidity) [ 13 ]. The absence of a significant difference in the penetration depth of the sealer between the PAH and PBeta groups can be related to the use of plasma that causes the drying of the dentin and the collapse of dentinal tubules [ 19 ]. In this study, there was a significant difference in the penetration depth of the sealer only in the coronal sections in AH and Beta groups, which might be due to the greater number of dentinal tubules and the easier penetration of the sealer at the coronal sections [ 20 ]. Finally, a positive opinion can be given about the Beta sealer that was recently introduced in terms of the depth of penetration due to the lack of significant difference in the depth and penetration percentage in the study groups that were exposed to plasma and the observation of a significant difference only in the coronal section in the groups that were not exposed to plasma. As for the limitations of the study, we can mention the difficulty of providing samples that match the inclusion and exclusion criteria of our study design.

## Conclusion

The use of plasma does not affect the maximum penetration depth and percentage of AH and Beta sealers.
